# (–)-Epicatechin Modulates Mitochondrial Redox in Vascular Cell Models of Oxidative Stress

**DOI:** 10.1155/2020/6392629

**Published:** 2020-06-09

**Authors:** Amy Keller, Sara E. Hull, Hanan Elajaili, Aspen Johnston, Leslie A. Knaub, Ji Hye Chun, Lori Walker, Eva Nozik-Grayck, Jane E. B. Reusch

**Affiliations:** ^1^Division of Endocrinology, Metabolism & Diabetes, University of Colorado Anschutz Medical Campus, Aurora, CO 80045, USA; ^2^Rocky Mountain Regional VA Medical Center, Aurora, CO 80045, USA; ^3^Cardiovascular Pulmonary Research Laboratories and Pediatric Critical Care, University of Colorado Anschutz Medical Campus, Aurora, CO 80045, USA; ^4^Division of Cardiology, University of Colorado Anschutz Medical Campus, Aurora, CO 80045, USA

## Abstract

Diabetes mellitus affects 451 million people worldwide, and people with diabetes are 3-5 times more likely to develop cardiovascular disease. In vascular tissue, mitochondrial function is important for vasoreactivity. Diabetes-mediated generation of excess reactive oxygen species (ROS) may contribute to vascular dysfunction via damage to mitochondria and regulation of endothelial nitric oxide synthase (eNOS). We have identified (–)-epicatechin (EPICAT), a plant compound and known vasodilator, as a potential therapy. We hypothesized that mitochondrial ROS in cells treated with antimycin A (AA, a compound targeting mitochondrial complex III) or high glucose (HG, global perturbation) could be normalized by EPICAT, and correlate with improved mitochondrial dynamics and cellular signaling. Human umbilical vein endothelial cells (HUVEC) were treated with HG, AA, and/or 0.1 or 1.0 *μ*M of EPICAT. Mitochondrial and cellular superoxide, mitochondrial respiration, and cellular signaling upstream of mitochondrial function were assessed. EPICAT at 1.0 *μ*M significantly attenuated mitochondrial superoxide in HG-treated cells. At 0.1 *μ*M, EPICAT nonsignificantly increased mitochondrial respiration, agreeing with previous reports. EPICAT significantly increased complex I expression in AA-treated cells, and 1.0 *μ*M EPICAT significantly decreased mitochondrial complex V expression in HG-treated cells. No significant effects were seen on either AMPK or eNOS expression. Our study suggests that EPICAT is useful in mitigating moderate ROS concentrations from a global perturbation and may modulate mitochondrial complex activity. Our data illustrate that EPICAT acts in the cell in a dose-dependent manner, demonstrating hormesis.

## 1. Introduction

Diabetes mellitus (DM) confers an excess risk of cardiovascular disease (CVD), preceded by dysfunction in vascular reactivity [1]. In the context of DM, excess reactive oxygen species (ROS) correlate with vascular inflammation and vascular stiffness [2–4]. Disruptions in redox regulation and elevated ROS are linked to hyperglycemia, dampened antioxidant defenses, insulin resistance, and dysfunctional cellular signaling [2, 3, 5–8]. It is established that elevated ROS promotes vascular pathology; however, multiple clinical attempts at establishing the efficacy of antioxidants have failed [9, 10]. Stochiometric approaches to excess ROS alleviation do not consider the important signaling role of ROS in cellular homeostasis [11, 12]. Therefore, studies targeting elevated ROS must approach redox from a broad perspective. For example, mitochondria are a central source of cellular superoxide under normal physiological conditions; however, excess mitochondrial-derived oxidative damage has been shown to cause age-related vascular inflammation and arterial stiffness [13–16]. As the mitochondrial function is critical to effective vasoreactivity, targeting redox homeostasis in this organelle is a promising therapeutic direction.

Flavonoids are a class of botanical compounds found ubiquitously in common plant-based foods that impact vasomotion. Flavonoids are characterized by a two-benzene ring basal structure and include well-known subclasses of compounds such as anthocyanins and catechins; as a class, these chemicals promote vasodilation and have specific antioxidant activity [17, 18]. The botanical flavonoid (–)-epicatechin (EPICAT) is found in commonly consumed foods, primarily chocolate (*Theobroma cacao*, Sterculiaceae) and tea (*Camellia sinensis*, Theaceae). This compound has been shown to induce vasorelaxation in rat femoral artery ex vivo, increase mitochondrial respiration in cardiomyocytes, prevent derangements in mitochondrial membrane potential and decrease in mitochondrial complex expression in mouse kidney cells, and activate nitric oxide synthase (NOS) activity, the enzyme upstream of both vasodilation and mitochondrial activity, in rat aorta and human coronary arterial endothelial cells [19–23].

Importantly, EPICAT has been reported to have antioxidant activity in multiple tissues and cells. Specifically, EPICAT supports the activation of ROS-regulating transcription factors and decreases ROS in aortic rings and HepG2 cells, measured with immunohistochemistry (IHC) and dihydroethidium (DHE) [24, 25]. EPICAT inhibits cardiac, hepatic, adipose, and HepG2 ROS-generating enzymatic or protein expression activity, such as NADPH oxidases (NOXs) and related proteins [26–28]. EPICAT has also been shown to alleviate hydrogen peroxide production in damaged cardiac and brain mitochondria by modifying mitochondrial respiration [29]. In plasma and urine, EPICAT promotes the activity of superoxide dismutase (SOD) and glutathione peroxidase [30]. In human lung fibroblasts, EPICAT restored SOD activity and complex I expression dampened by damaged mitochondria [31]. Despite this consistent antioxidant paradigm in numerous models and tissues, EPICAT's mechanism(s) at the cellular level remain largely unverified.

We hypothesized that in a vascular cell model, human umbilical vein endothelial cells (HUVECs), treated with antimycin (mitochondrial perturbation, AA) or high glucose (cellular perturbation, HG) will generate mitochondrial ROS and that treatment with EPICAT will reduce mitochondrial ROS concentrations while not impacting untreated cells, restoring mitochondrial function and cellular homeostasis. We chose these two cellular perturbations to test EPICAT against oxidative stress targeted to the mitochondria as well as global cellular stress. It is imperative to have these two models, as EPICAT has mixed results as an antioxidant. Employing a mitochondrial specific versus global model allows for specific determination about its activity. Here, we report our results from testing concentrations of 0.1 or 1.0 *μ*M of EPICAT, based on previous literature, in cells exposed to either AA or HG. We measured cytosolic or mitochondrial-derived superoxide, mitochondrial respiration, and cellular nutrient signaling, as well as endogenous redox defenses. Our study demonstrates the dose-dependent redox and mitochondrial regulatory activity of EPICAT. This effect is of interest for diseases characterized by chronic redox dysfunction impacting the vasculature, such as diabetes. To our knowledge, this is a singular, comprehensive effort to investigate EPICAT's bioactivity in a human vascular cell model with the goal of expanding the understanding of the actions of this compound beyond its well-established activity as a vasodilator.

## 2. Methods and Materials

### 2.1. Reagents

For cell culture, Hyclone Ham's Nutrient Mixture F12 Media (Fisher #SH30526.01) was purchased from Fisher. Penicillin/streptomycin, trypsin, and fetal bovine serum (FBS) were purchased from Gemini Bioproducts (CA, USA). For Western blotting and general experiments, gels were from BioRad, PVDF membranes from Millipore, fluorescent secondary antibodies were from Licor. Mammalian Protein Extraction Reagent (M-PER,) was obtained from Thermo Scientific Hyclone (MA, USA), and dimethyl sulfoxide (DMSO), sodium chloride, sucrose, and bovine serum albumin were purchased from Fisher Scientific (PA, USA). For Western blot and respiration experiments, collagenase, ethylenediaminetetraacetic acid (EDTA), ethylene glycol tetraacetic acid (EGTA), sodium pyrophosphate, sodium orthovanadate, sodium fluoride, okadaic acid, 1% protease inhibitor cocktail, dithiothreitol, magnesium chloride, K-lactobionate, taurine, potassium phosphate, HEPES, digitonin, pyruvate, malic acid, glutamic acid, adenosine diphosphate, succinic acid, oligomycin, carbonyl cyanide 4 (trifluoromethoxy)phenylhydrazone (FCCP), antibody to *β*-actin (mouse), phenylephrine and acetylcholine, trypsin inhibitor, and cytochrome c were procured from Sigma-Aldrich (MO, USA). EPICAT was sourced from Cayman Chemical (MI, USA).

### 2.2. Antibodies

Antibodies to total adenosine monophosphate kinase (AMPK, Cell Signaling #2532S, 1 : 500, mouse), phosphorylated AMPK (pAMPK, Cell Signaling #2532S, 1 : 500, rabbit), Sirtuin 3 (SIRT3, Cell Signaling #2627S, 1 : 500, rabbit), total endothelial nitric oxide synthase (eNOS, Cell Signaling #9572S, 1 : 500-1 : 250, mouse), Ser1177 phosphorylated eNOS (Cell Signaling #9571S, 1 : 500-1 : 250 rabbit), were obtained from Cell Signaling (MA, USA). Antibody cocktail to representative subunits of mitochondrial oxidative phosphorylation (Total OXPHOS Blue Native WB Antibody Cocktail Abcam #ab110412, 1 : 1000-1 : 500, mouse) complexes I (subunit NDUFA9), II (subunit SDHA), III (subunit UQCRC2), IV (subunit IV), and V (subunit ATP5A); PPAR*γ* coactivator 1 alpha (PGC-1*α*, Abcam #ab54481, 1 : 500, rabbit); and MnSOD antibody (Anti-SOD2/MnSOD antibody [2a1], Abcam, #ab16956, 1 : 1000-1 : 500) were obtained from Abcam (Cambridge, MA). Secondary Fluorescent antibodies (IRDye 680RD goat antimouse, Li-COR, #926-68070 1 : 5,000, IRDye 680RD goat antirabbit, Li-COR, #926-68071 1 : 5,000, IRDye 800CW goat antimouse, Li-COR, #926-32210, 1 : 10,000, IRDy 800CW goat antirabbit, Li-COR, #926-32211, 1 : 10,000) for Western blot detection were purchased from Li-COR (NE, USA).

### 2.3. Cell Experiments

Human umbilical vein endothelial cells (HUVECs) were purchased from ATCC and grown in media supplemented with 10% FBS and 1% penicillin/streptomycin at 7 mM glucose. Cells were incubated in 0.1% FBS starvation media for 12-15 hours. HUVECs were then preincubated for 1 hour with either 0.1 or 1.0 *μ*M EPICAT diluted into phosphate-buffered saline (PBS) or PBS alone. Following this incubation, antimycin (10 *μ*M, AA), ethanol, glucose (30 mM, high glucose [HG]), or PBS were directly added into media for a 2-hour incubation. The antimycin concentration was chosen based on previous studies of mitochondrial ROS generation [32, 33]. Cells were then harvested for electron paramagnetic resonance spectroscopy, respiration, or Western blotting. No evidence of cell death was observed. All experiments were conducted in triplicate or quadruplicate.

### 2.4. Electron Paramagnetic Resonance Spectroscopy (EPR)

Total ROS production was measured by EPR using the superoxide sensitive spin probe 1-hydroxy-3-methoxycarbonyl-2,2,5,5-tetramethylpyrrolidine (CMH), while mitochondrial ROS production was measured using the mitochondrial spin probe 1-hydroxy-4-[2-triphenylphosphonio)-acetamido]-2,2,6,6-tetramethyl-piperidine,1-hydroxy-2,2,6,6-tetramethyl-4-[2-(triphenylphosphonio)acetamido] piperidinium dichloride (mito-TEMPO-H). HUVEC Cells were seeded in 6-well plates, and experiments were completed prior to the EPR measurements. Spin probes CMH and mito-TEMPO-H were prepared in deoxygenated 50 mM phosphate buffer. Cells were washed and treated with CMH and mito-TEMPO-H 0.25 mM in Krebs-HEPES buffer (KHB) containing 100 *μ*M of a metal chelator DTPA. Cells were incubated for 50 min at 37°C then gently scraped and transferred to ice. 50 *μ*l of cell suspension was loaded in an EPR capillary tube, and EPR measurements were performed at room temperature using Bruker EMXnano X-band spectrometer. EPR acquisition parameters are microwave frequency = 9.6 GHz; center field = 3432 G; modulation amplitude = 2.0 G; sweep width = 80 G; microwave power = 19.9 mW; total number of scans = 10; sweep time = 12.11 s; and time constant = 20.48 ms. CMH or mito-TEMPO-H both are detected as nitroxide radicals; the concentration was obtained by simulating the spectra using the SpinFit module incorporated in the Xenon software of the bench-top EMXnano EPR spectrometer followed by the SpinCount module (Bruker). Nitroxide concentrations were normalized to total protein.

### 2.5. Western Blotting

HUVECs were harvested in 4°C mammalian lysis buffer (MPER with 150 mM sodium chloride, 1 mM of EDTA, 1 mM EGTA, 5 mM sodium pyrophosphate, 1 mM sodium orthovanadate, 20 mM sodium fluoride, 500 nM okadaic acid, 1% protease inhibitor cocktail), and protein was measured using Western blotting as previously described [34]. Cell lysates were sonicated at 4°C centrifuged at 18,000 x g at 4°C for 10 min, and the Bradford protein assay was used to measure the protein concentration of the lysate. Protein samples (15 *μ*g to 40 *μ*g) in Laemmli sample buffer (boiled with 100 mM dithiothreitol) were run on precast SDS-4-15% polyacrylamide gels. Proteins were transferred to PVDF membranes. Ponceau S staining was used to evaluate protein loading. Blots were probed with antibodies described above and left overnight at 4°C. Fluorescent secondary antibodies were applied following the primary antibody incubation (1 : 10,000 IRDye800CW and 1 : 5,000 IRDye680RD, 1 hour at room temperature). Proteins were detected by fluorescence with the Li-COR Odyssey CLX, and Image Studio v 4.1 was used for densitometric analysis. All protein data has been normalized to *β*-actin protein expression. Specific activity was determined as the ratio of phosphorylated signal to total signal following *β*-actin normalization. To rule out bleed-through, antibodies were probed on the same blot using different animal primary antibodies between the phosphorylated (rabbit) and total protein (mouse) allowing for two-color detection and analysis when used with secondary fluorescent antibodies with differing wavelengths (IRDye 680RD and IRDye 800CW).

### 2.6. Respiration

Oroboros Oxygraph-2k (O2k, OROBOROS INSTRUMENTS Corp., Innsbruck, Austria) was used for mitochondrial respiration analysis. Permeabilized HUVEC protocols were optimized according to previously described protocols [34–36]. HUVECs were trypsinized using 0.25% trypsin/EDTA, washed with PBS, and spun (3 minutes at 800 g). Cells were then resuspended in MiR05 respiration buffer (0.5 mM EGTA, 3 mM magnesium chloride, 60 mM K-lactobionate, 20 mM taurine, 10 mM potassium phosphate, 20 mM HEPES, 110 mM sucrose, 1 g/l fatty acid-free bovine serum albumin) and counted under a microscope using a hemocytometer. HUVECs were added to the O2k chamber at a cell count of 0.5 × 10^6^ and 1 × 10^6^ per chamber and permeabilized with 3 *μ*g of digitonin. Substrates and inhibitors designed to mimic carbohydrate metabolism were added to assess respiration rates. State 2 (leak state) was defined following the addition of 5 mM pyruvate, 2 mM malate, and 10 mM glutamate (PMG); state 3 (ATP-generating respiration) was defined as PMG with 2 mM adenosine diphosphate (ADP); state 3S was defined as PMG, ADP, and 6 mM succinate; 2 *μ*g/ml oligomycin revealed state 4 (leak state); and 0.5 *μ*M of carbonyl cyanide 4-(trifluoromethoxy)phenylhydrazone (FCCP) was added incrementally until a peak uncoupling state was reached (uncoupled). Cells were recounted following the experiments, and respiration rates normalized to cell count. An area of consistent respiration rate of 3-5 minutes or longer was representative of the various states.

### 2.7. Statistical Analysis

A two-way ANOVA was used for data analysis with Tukey multiple comparisons post hoc analysis for comparing each group. Data are presented on separate graphs to represent each ANOVA comparison. For analysis of the mitochondrial superoxide data, controls (negative and positive for both HG and AA) from all experiments were pooled. A *p* value of less than 0.05 for interaction, treatment, or EPICAT effects was used as the cutoff for statistical significance in all tests. A *p* value of equal or less than 0.08 was considered indicative of data trends approaching significance. Data are expressed as mean ± SEM.

## 3. Results

### 3.1. Differential Measurement of Total Cellular Versus Mitochondrial Superoxide

**Figure 1:**
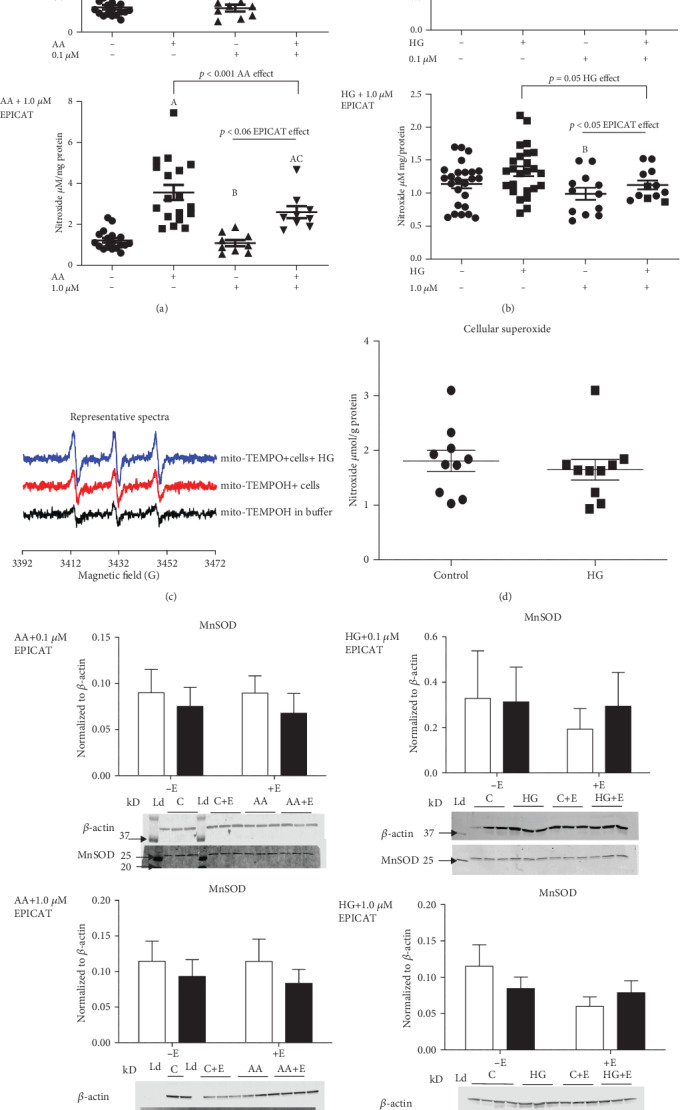
(a–d) Mitochondrial and cellular superoxide measurement in both HG and AA perturbations: Superoxide concentrations were measured in HUVECs by electron paramagnetic resonance spectroscopy using two different spin probes to differentiate mitochondrial (Mito-TEMPO-H) and total cell (CMH) superoxide. CM or mito-TEMPO nitroxide radicals concentration was obtained by simulating the spectra using the SpinFit module incorporated in the Xenon software of the bench-top EMXnano EPR spectrometer followed by the SpinCount module (Bruker). Nitroxide concentrations were normalized to total protein. Mitochondrial superoxide (a–c) and MnSOD protein expression (e) was assessed in cells exposed to 0.1 *μ*M and 1.0 *μ*M EPICAT *n* = 3 − 4, in control, and HG- and AA-treated cells. Representative spectra is shown (c), and total cellular superoxide during a HG perturbation is shown (d), *n* = 3. For analysis of superoxide, control data from all experiments was pooled *n* = 8, and separate tests were run on each experiment of different EPICAT concentrations. AA experiments, *p* < 0.001 AA effect, both experiments, †*p* = 0.06 EPICAT effect for 1.0 *μ*M concentration only (a). HG experiments, *p* = 0.05 glucose effect, ∗*p* < 0.05 EPICAT effect, 1.0 *μ*M concentration only (b), two-way ANOVA, Tukey multiple comparisons analysis. A long horizontal bar over the entire graph indicates an interaction effect, smaller bars over the EPICAT groups indicates an EPICAT effect, while bars with tabs indicate a single main effect of either AA or HG. Post hoc analyses are described as *a* = *p* < 0.05 as compared to control, *b* = *p* < 0.05 as compared to treatment, *c* = *p* < 0.05 as compared to EPICAT control. Data are expressed as mean ± SEM.

We employed electron paramagnetic resonance spectroscopy using two different spin probes to differentiate total cell (CMH) and mitochondrial (Mito-TEMPO-H) superoxide to measure both total cellular and mitochondrial-derived superoxide. When the mitochondrial probe was used in these same experiments, we measured elevated superoxide concentrations in both AA (*p* = 0.05, Figure 1(a)) and HG-treated cells (*p* < 0.05, Figure 1(b)). Representative nitroxide spectra for mitochondrial-specific superoxide are shown in Figure 1(c). To determine whether we could isolate the measurement of mitochondrial-derived superoxide from total cellular superoxide, we tested cells with HG and measured superoxide in total cells (Figures 1(a) and 1(d)). HG failed to generate significantly higher concentrations of superoxide in the total cell as compared with the control (Figure 1(d)), demonstrating the mitochondrial specificity of our superoxide measurements.

### 3.2. (–)-Epicatechin Attenuated Mitochondrial Superoxide Production

In all experiments, the mitochondrial toxin and superoxide generator AA significantly increased superoxide production (*p* < 0.05, Figure 1(a)). AA plus EPICAT at 1.0 *μ*M (Figure 1(a)) tended to decrease superoxide in cells compared to AA alone (*p* = 0.06, EPICAT effect, Figure 1(a)). Post hoc analysis revealed that AA-treated cells showed significantly elevated superoxide as compared with controls, and cells with control plus EPICAT at both concentrations had significantly lower superoxide as compared with treated cells (*p* < 0.05, Figure 1(a)). Cells with AA plus EPICAT were significantly different than both controls with and without EPICAT (both concentrations, *p* < 0.05, Figure 1(a)). At 1.0 *μ*M, EPICAT significantly attenuated superoxide production stimulated by HG, with post hoc analysis revealing significantly less superoxide in cells with EPICAT as compared with HG-treated cells (*p* = 0.05 and *p* < 0.05, interaction and EPICAT effects, respectively, Figure 1(b)). EPICAT at 0.1 *μ*M concentrations did not show any significant impact on mitochondrial-derived superoxide (Figure 1(b)). MnSOD expression was not altered by either AA or HG, alone or in the presence of EPICAT treatment (Figure 1(e)).

### 3.3. (–)-Epicatechin Had No Significant Impact on Mitochondrial Respiration

**Figure 2:**
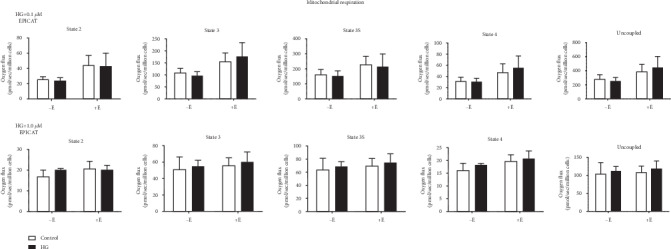
Mitochondrial respiration of permeabilized cells. Permeabilized HUVECs were exposed to substrates and inhibitors mimicking carbohydrate metabolism and states 2 (leak state), 3, 3S (both ATP-generating respiration), 4 (leak state), and uncoupled were determined. Respiration rates normalized to cell count (*n* = 4). ∗*p* < 0.05, †*p* < 0.08, interaction, treatment, or EPICAT effect, two-way ANOVA, Bonferroni's multiple comparisons analysis. Data are expressed as mean ± SEM.

Permeabilized HUVECs were exposed to a suite of substrates and inhibitors designed to mimic carbohydrate metabolism; respiration was measured as mitochondrial oxygen disappearance. No significant differences were observed in any respiration state in response to HG and EPICAT treatment (Figure 2). However, a nonsignificant increase in all respiration states was observed with 0.1 *μ*M EPICAT (Figure 2). Cells treated with AA showed decreased respiration at all states, as expected with a mitochondrial toxin; however, the toxic effect of AA did not permit full respiration measurements (data not shown).

### 3.4. (–)-Epicatechin Modulated AMPK Expression

**Figure 3:**
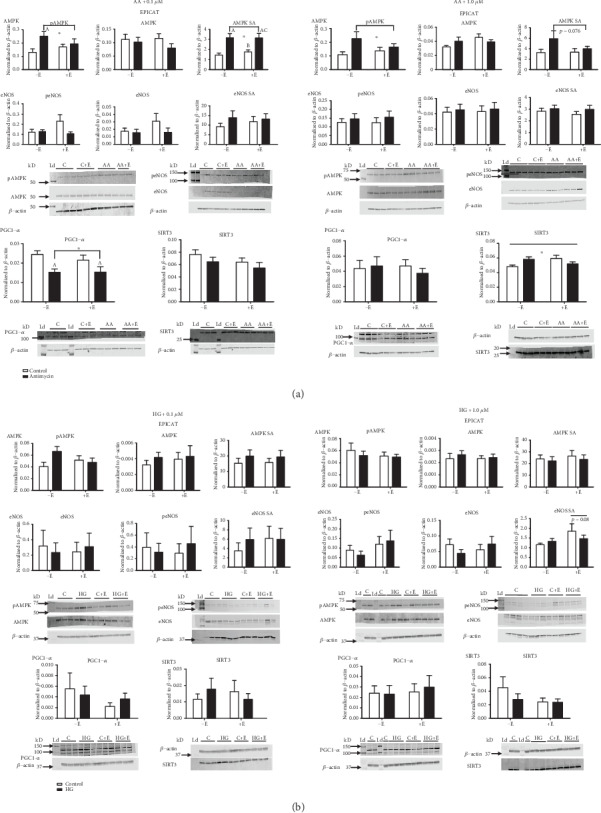
(a, b) Cellular signaling: Cells were harvested and lysates processed for protein expression via Western blot analysis (*n* = 3 − 4). Blots were probed for pAMPK, AMPK, peNOS, eNOS, SIRT3, and PGC1-*α*. Specific activity (SA) was calculated as phosphorylated signal normalized to total signal for AMPK and eNOS. ∗*p* < 0.05, interaction, treatment, or EPICAT effect, two-way ANOVA, Tukey multiple comparisons analysis. A long horizontal bar over the entire graph indicates an interaction effect, smaller bars over the EPICAT groups indicates an EPICAT effect, while bars with tabs indicate a single main effect of either AA or HG. Post hoc analyses are described as *a* = *p* < 0.05 as compared to control, *b* = *p* < 0.05 as compared to treatment, *c* = *p* < 0.05 as compared to EPICAT control. Data are expressed as mean ± SEM.

AA treatment resulted in a significant increase in pAMPK expression, regardless of EPICAT concentrations (*p* < 0.05, Figure 3(a)). AA treatment also elevated AMPK specific activity at 0.1 *μ*M EPICAT (*p* < 0.01, Figure 3(a)). Post hoc analysis revealed significant differences in pAMPK expression between control and treated cells in the 0.1 *μ*M EPICAT experiments, and also in AMPK specific activity between control cells plus EPICAT and AA-treated cells, and between control and AA-treated cells with EPICAT (*p* < 0.05, Figure 3(a)). Post hoc analyses showed no differences between groups in the 1.0 *μ*M EPICAT experiments (Figure 3(a)). In cells treated with AA, EPICAT did not impact pAMPK expression (Figure 3(a)). In cells treated with HG, no effects were noted on pAMPK expression (Figure 3(a)).

### 3.5. (–)-Epicatechin Nonsignificantly Impacted eNOS Activity

In cells treated with AA, 0.1 *μ*M EPICAT resulted in no change in peNOS expression (Figure 3(a)). In cells treated with HG, 1.0 *μ*M EPICAT resulted in a nonsignificant elevation of eNOS specific activity (*p* = 0.08, Figure 3(b)).

### 3.6. (–)-Epicatechin Modulated the Expression of SIRT3 but Did Not Impact PCG1-*α*

Cells exposed to AA showed a significant decrease of PGC1-*α* expression (*p* < 0.01, treatment effect and significant post hoc differences between control and AA-treated cells, *p* < 0.05, Figure 3(a)). Post hoc analysis revealed significant differences between both AA-treated groups and the control cells (*p* < 0.05, Figure 3(a)). No effect of EPICAT was observed. A significant interaction effect between treatment and EPICAT was observed in SIRT3 expression in HUVECs treated with AA and 1.0 *μ*M EPICAT (*p* < 0.01, interaction effect, *p* < 0.05 difference between vehicle control ± EPICAT, Figure 3(a)); however, no differences were noted in post hoc analyses. No effects were noted with HG-treated cells (Figure 3(b)).

### 3.7. (–)-Epicatechin Attenuated the Expression of Mitochondrial Complexes I and V

**Figure 4:**
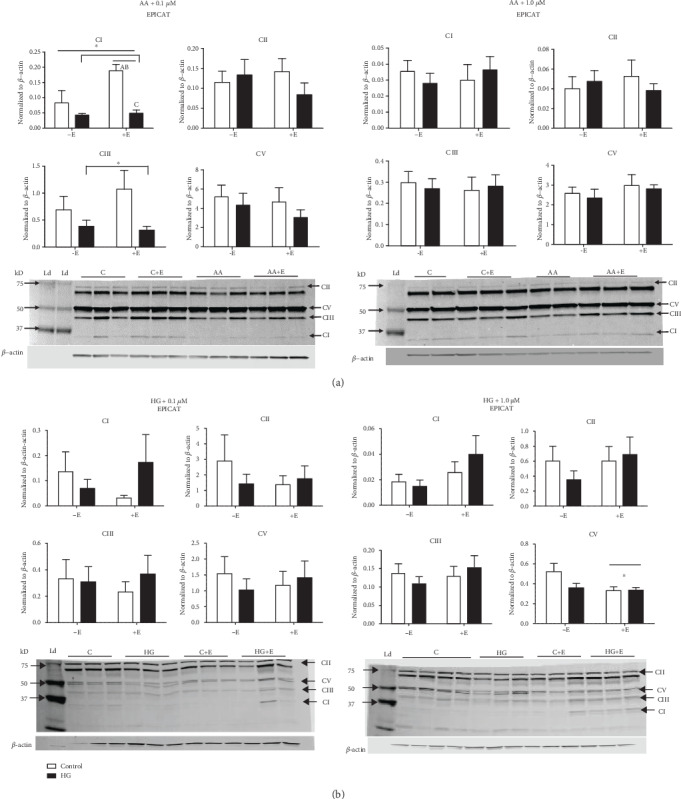
(a, b) Mitochondrial complex expression: Cells were harvested and lysates processed for protein analysis via Western blot analysis (*n* = 3 − 4). Blots were probed for mitochondrial complexes I, II, III, and IV using a single antibody-containing subunits of all complexes. ∗*p* < 0.05, †*p* < 0.08, interaction, treatment, or EPICAT effect, two-way ANOVA, Tukey multiple comparisons analysis. A long horizontal bar over the entire graph indicates an interaction effect, smaller bars over the EPICAT groups indicates an EPICAT effect, while bars with tabs indicate a single main effect of either AA or HG. Post hoc analyses are described as *a* = *p* < 0.05 as compared to control, *b* = *p* < 0.05 as compared to treatment, *c* = *p* < 0.05 as compared to EPICAT control. Data are expressed as mean ± SEM.

In cells exposed to AA, there was a significant decrease in complex I expression with 0.1 *μ*M EPICAT (interaction (*p* < 0.05), treatment (*p* < 0.01), and EPICAT effect (*p* < 0.05), with significant post hoc differences between control cells with EPICAT and both control and AA-treated cells, and between control and AA-treated cells with EPICAT *p* < 0.05 for all, Figure 4(a)). Complex III expression was significantly decreased by AA treatment (*p* < 0.05, treatment effect, Figure 4(a)). In HUVECs exposed to HG, EPICAT at 1.0 *μ*M concentration significantly attenuated the expression of mitochondrial complex V (*p* < 0.05, EPICAT effect, *p* < 0.05, significant differences between vehicle control ± EPICAT, Figure 4(b)). No impact on complex expression was observed with 0.1 *μ*M concentration (Figure 4(b)).

## 4. Discussion

Here, we report our results from an acute in vitro study investigating the bioactivity of two concentrations of EPICAT in vascular cells. Endothelial cells were exposed to insults designed to mimic either direct mitochondrial insult (AA) or broad metabolic stress (HG). These perturbations were specifically designed to gauge the biological activity of EPICAT at the cellular level, harnessing HUVEC's, a human endothelial cell, versatility for in vitro vascular studies. The dosage range of EPICAT 0.1-1.0 *μ*M has been used extensively in the literature, particularly in in vitro experiments [21, 37]. At these concentrations in cells, EPICAT attenuated mitochondrial superoxide in HG-treated cells but not in AA-treated cells, stimulated a nonsignificant mitochondrial respiration signal, and modulated mitochondrial complex expression. Although others have reported an impact of EPICAT on cellular signaling, we do not observe this in our acute study at 2 hours of treatment.

To date, very little research has been conducted in an informative vascular cell model to provide a detailed understanding of the vasoreactivity results observed with EPICAT in vivo. Using a comprehensive suite of endpoints to pinpoint EPICAT bioactivity on mitochondrial ROS and content and function, we found a difference between the response of a mitochondrial poison (AA) versus nutrient stress (HG) on ROS profiles and mitochondrial respiration using state of the art approaches. EPR enables precise and specific superoxide measurement to delineate superoxide concentrations of mitochondrial origin. We chose our cell model, HUVECs, as they are a human-derived in vitro model widely used in vascular cellular studies. A 2-hour incubation period was chosen based on preliminary experiments addressing specific intercellular ROS pools, mimicking the acute postmeal state. The novelty of our current study lies, in part, in its assessment of cellular signaling and bioactivity upstream of vasodilation during an acute AA (targeted) or HG (global) exposure.

### 4.1. (–)-Epicatechin Suppresses Mitochondrial Superoxide

Here, we observed a suppression of AA-induced mitochondrial superoxide at the higher concentration of EPICAT, albeit not significant, and significant lowering effect of EPICAT on HG-induced superoxide, confirming previous studies suggesting that EPICAT is an antioxidant [22, 24–31, 38]. We also demonstrate that both AA and HG specifically increase mitochondrial ROS acutely. Surprisingly, we did not observe a concurrent response in mitochondrial superoxide dismutase (MnSOD); however, we did capture significant elevation of SIRT3 expression with 1.0 *μ*M EPICAT concentration in our AA perturbation experiments. EPICAT has previously been shown to have antioxidant activity [22, 38], but the mechanisms of this activity are not entirely elucidated. Taken together, our data strongly suggest that EPICAT does not work stoichiometrically, as has been seen with other compounds such as vitamin C [10, 11], but modulates endogenous cellular redox defenses. These data show EPICAT as an intriguing solution to excess mitochondrial superoxide, a known phenomenon in chronic disease, such as diabetes and metabolic syndrome [2, 3, 5–8].

### 4.2. (–)-Epicatechin Modulated Mitochondrial Activity

EPICAT has been shown to increase mitochondrial respiration [23, 39]. However, we did not observe any significant impact on mitochondrial respiration at 2 hours. Interestingly, EPICAT treatment at the higher concentration resulted in a significantly less expression of complex V in cells treated with HG; this may indicate a dose-dependent impact of EPICAT depressing mitochondrial function and therefore lessening the generation of superoxide. At the AA experiment with 0.1 *μ*M EPICAT treatment, EPICAT significantly increased complex I expression in control cells but failed to rescue the AA-dampened response in AA-treated cells. EPICAT also failed to restore complex III expression dampened in AA-treated cells. This suggests a stimulatory effect of EPICAT on mitochondrial activity that is not sufficient to overcome the toxicity caused by AA. This dampening of complex expression is expected in AA-treated cells, as AA targets complex III of the electron transport chain. Employing the use of the Oxygraph 2k Oroboros in conjunction with protein expression measurements is a highly rigorous way to assess mitochondrial function; here, we show that EPICAT may stimulate mitochondrial respiration while protecting against resultant oxidant damage, but cannot restore cell function in the context of a specific mitochondrial insult.

### 4.3. (–)-Epicatechin Has Only a Moderate Effect on Cellular Signaling Upstream of Mitochondria Regulation

EPICAT has been shown to increase or modulate AMPK and eNOS signaling in previous studies [20, 21, 40, 41]. Our results show that AA had a significant stimulatory effect on AMPK expression, but EPICAT was unable to modulate this effect. Our results may not agree with those previously reported due to the acute nature of our study (2 hours of incubation with AA or HG), or other experimental differences. Other studies report that EPICAT induces and increases eNOS expression, but we failed to see a significant increase in the eNOS expression or specific activity in either AA- or HG-treated cells. We acknowledge that we measured protein expression, not a true enzymatic activity for either pAMPK or eNOS. We anticipated that the impact on cellular signaling would be detectable after 2 hours of perturbation. Further studies will be needed to determine whether EPICAT signals more acutely, whereas longer incubations may result in indirect effects. Further in vitro experiments in other vascular cell lines and experimental designs may clarify the activity of these and additional mechanisms of action of EPICAT in cellular signaling.

### 4.4. Hormesis

Our data largely demonstrate that EPICAT is more bioactive at the lower concentration tested as compared with the higher concentration. This phenomenon, known as hormesis, refers to a biphasic response of beneficial stimulation of cellular response at low doses, but toxic or null activity promoted by compounds at higher concentrations [42]. Hormesis is observed with many phytochemicals, including catechins [43], and the overall result is homeostatic adaptation [42]. This concept agrees with other reports of a peak bioactive EPICAT concentration for the stimulation of mitochondrial complex expression in vitro, with higher concentrations showing diminished bioactivity [44, 45]. This observation of EPICAT's hormetic effect is also reported in certain hormones sharing a common structural backbone [45], perhaps explaining this commonality in demonstrating hormesis. Taken together, these results show that EPICAT is an agent of hormesis, ultimately promoting cellular and physiological adaptation. These results also agree with a recent paradigm shift on the role of ROS and antioxidants in health [46, 47]. Current understanding on mitochondrial-derived ROS considers the possibility that this excess mitochondrial ROS may have a hormetic effect on cellular pathways in the context of chronic glucose exposure, providing cellular adaptation to nutrient excess [47]. Our results showing bioactivity at a lower dose of EPICAT, and those of others reporting lack of clinical impact of antioxidant supplements [46], align with this new paradigm. It is possible that EPICAT has an adaptive effect on cellular homeostasis at a low concentration but allows for cells' necessary responsiveness to higher ROS concentrations of mitochondrial-derived ROS.

### 4.5. Limitations

Previous preliminary and scout experiments in our laboratory pointed to the acute bioactivity of EPICAT, impacting downstream cellular activity in incubation periods shorter than 4 hours. Two-hour incubations were chosen to determine whether EPICAT works acutely and signals at the cellular level. As most of our cellular signaling and respiration data failed to yield significant activity, longer incubation periods may be necessary in future experiments. We did not measure enzymatic activity directly; we assessed protein expression, a proxy measurement. Also, we chose the HUVECs as a widely used model of vascular cells; however, these cells are venous in origin, and using cells from arteries may be more representative of vascular physiology. Lastly, we report many endpoints showing only nonsignificant results (0.05 ≤ *p* ≤ 0.08). We are confident that we have repeated our experiments sufficiently; thus, this may be due to reasons stated above.

## 5. Conclusions

Our study shows that EPICAT acutely supports cellular homeostasis in the context of oxidative stress, but not cellular signaling. EPICAT is found in several commonly consumed foods, such as chocolate and tea, and although we made no attempt to compare our dosage in vitro with that of edible EPICAT-containing sources, future studies will ideally investigate this further. Our future experiments will elucidate the mechanism behind EPICAT's redox normalization and modulation of mitochondrial regulation and as a potential therapeutic target. We also made use of an exciting tool (EPR) for the measurement of cellular superoxide. In conclusion, EPICAT shows promise as a potential modulator of oxidant stress that needs to be further studied in the setting of chronic ROS.

## Data Availability

Data will be provided upon request to the corresponding author.
